# Portable low-cost instrumentation for monitoring Rayleigh scattering from chemical sensors based on metallic nanoparticles

**DOI:** 10.1038/s41598-018-33271-8

**Published:** 2018-10-08

**Authors:** Glibver Vasquez, Yulán Hernández, Yves Coello

**Affiliations:** Departamento de Ciencias, Sección Química, Pontificia Universidad Católica del Perú PUCP, Lima, Peru

## Abstract

Using a Hg(II) sensor based on the aggregation of gold nanoparticles as a model system, we evaluated the performance of two portable low-cost devices that monitor the wavelength-ratiometric resonance Rayleigh scattering signal of the chemical sensor upon white-LED illumination. The first device uses two optical filter-photodiode combinations to detect scattered light while the second employs a novel ultra-compact (grating-free) spectral sensor. Results show that the response of the Hg(II) sensor monitored with these devices is comparable to that measured using a high-end benchtop scanning spectrofluorometer. The great potential of this new LED-spectral sensor was demonstrated with the quantification of Hg(II) in tap and spring water. Due to the promising results obtained, many reported chemical sensors based on Rayleigh scattering from metallic nanoparticles could take advantage of this compact portable instrumentation for cost-effective field-deployable applications.

## Introduction

In the past few decades, metallic nanoparticles have received much attention due to their optical, electrochemical and catalytic properties, which offer enormous opportunities for applications in various scientific and technical fields^1–3^. In particular, silver (AgNPs) and gold nanoparticles (AuNPs) have been widely employed in the design of chemical sensors for numerous relevant species in the medical, forensic, food safety and environmental fields, including proteins, DNA, toxins, and metallic ions. These chemical sensors offer excellent analytical performance (high sensitivity and selectivity) and rapid analysis times^4–10^.

Special attention has been paid to colorimetric chemical sensors based on AgNPs and AuNPs because of their high extinction (absorption plus scattering) cross-sections within their localized surface plasmon resonance (LSPR) band. The LSPR band results from the collective oscillations of conducting electrons and falls in the visible electromagnetic region for spherical AgNPs and AuNPs. The position of the LSPR band is affected by various factors such as the size and the shape of the nanostructures, their aggregation state and the environment where they are dispersed. These features explain why the wavelength or intensity of the LSPR band varies in the presence of different analyte concentrations. Thus, these plasmonic nanosensors can be monitored using simple visible spectrophotometers –or even by the naked eye although with lower limits of detection– circumventing the need for expensive instrumentation and time-consuming analysis required by conventional analytical techniques^4–10^.

Although significantly less explored than the colorimetric (extinction) detection scheme, resonance Rayleigh scattering (RRS) from metallic colloids can also be exploited in the design and implementation of chemical sensors^11,12^. RRS refers to the elastic scattering process produced when the frequency of the incident light is near an absorption band^13^. For 15 nm-AuNPs, the RRS intensity is comparable to that of fluorescence for the same optical density^11^. Interestingly, AgNPs display even higher RRS cross-sections as compared to AuNPs^11^ and have also been exploited in the design of RRS chemical sensors^14–16^ but suffer from rapid degradation which lowers their practical application. Furthermore, the RRS scattering efficiency increases as the nanostructure size grows, a very interesting feature for the detection of aggregation patterns^17^. Due to their high scattering cross-sections, these noble metal nanoparticles have even been used as single-particle scattering nanosensors both *in vitro* and *in vivo*^18,19^. In addition, metallic nanoparticles are extremely photostable as they do not photobleach or blink, unlike fluorophores. Based on these distinct advantages, a number of metallic colloid RRS chemical sensors have been reported for the detection of proteins^20^, cancer cells^21^, metallic ions^14,22–25^ and small molecules^12,14,26^. Most frequently, the sensor response is monitored by performing a monochromator scan in a benchtop instrument^12,14–16,21–26^. However, to be useful for field-deployable analyte quantification, the sensor needs to be monitored with a compact and portable device^27^.

Portable RRS chemical sensors based on metallic colloids could be exploited in a wide variety of fields including agriculture, environmental monitoring, forensics and even medicine. To demonstrate the potential of such a sensor, we developed an RRS Hg(II) nanosensor suitable for on-site water analysis. The quantification of Hg(II) in natural waters is relevant for environmental monitoring given the high toxicity of this heavy metal^28–32^, which poses a serious threat for the ecosystems and for public health, and is especially important in developing countries like Peru, where illegal gold mining is a major problem. The developed Hg(II) sensor was implemented using two portable low-cost devices capable of monitoring the wavelength-ratiometric RRS response of a chemical sensor based on metallic nanoparticles upon white-LED illumination. In contrast to a non-ratiometric RRS sensor, for which the scattering intensity at a single wavelength is monitored, the response of a wavelength-ratiometric RRS sensor is based on the ratio between the scattering intensities at two incident wavelengths, which makes the measurements independent of instrumental drift (illumination source or detector element fluctuations) and colloid concentration^11^.

## Results

### Ratiometric RRS Hg(II) sensor

**Figure 1:**
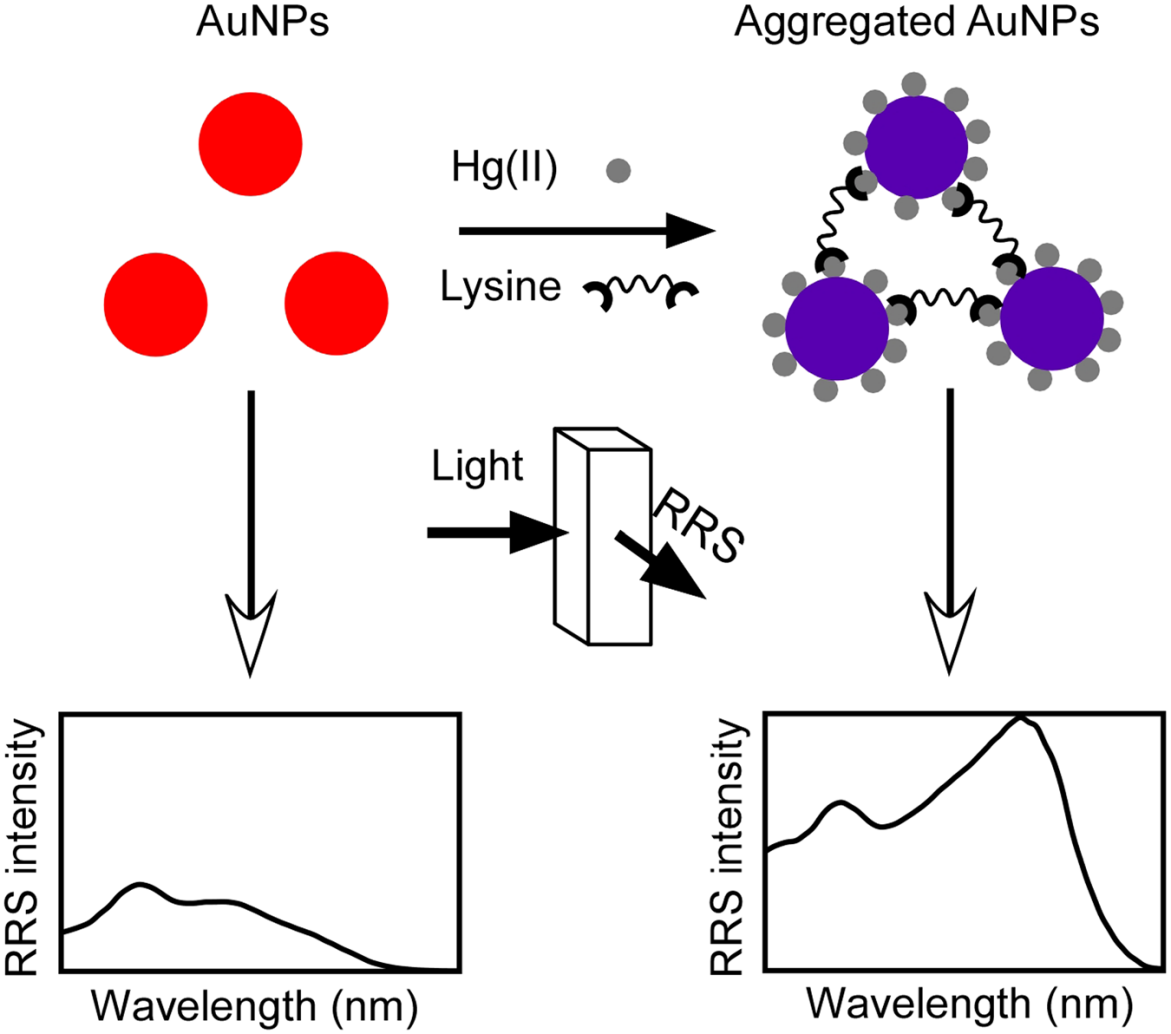
Schematic representation of the RRS Hg(II) sensing strategy. In the presence of Hg(II) and lysine as the aggregation promoter, Hg-covered AuNPs aggregate leading to an increase of the RRS response in the red region of the spectrum as a result of interparticle plasmonic coupling.

To develop the Hg(II) RRS nanosensor, we selected a colorimetric sensing strategy based on the aggregation of AuNPs promoted by lysine as a starting point^33^. This sensor is conveniently simple, as it works with as-prepared citrate-capped AuNPs, without requiring any surface modification of the colloid. The working mechanism is based on the affinity of the two amine moieties present in lysine toward Hg and involves two steps. First, Hg(II) is reduced by citrate on the surface of the AuNPs and then, lysine addition to the mixture induces aggregation of the Hg-covered AuNPs resulting in a fast red-to-purple color change as a result of interparticle plasmonic coupling (Fig. 1)^33,34^. Similarly to the reported colorimetric scheme, the RRS response of the sensor within the red region of the spectrum is expected to increase gradually due to the formation of AuNP aggregates^12^. A schematic representation of the Hg(II) sensing strategy is depicted in Fig. 1. The RRS detection scheme has the additional advantage of being able to be monitored with the simple and low-cost instrumentation described here.

As pointed out before, the RRS efficiency of a gold colloid increases with nanoparticle size^17^, although bigger AuNPs involve a more complex preparation and exhibit lower stabilities and monodispersity^12^. Here, we selected ~30 nm AuNPs seeking for a compromise between RSS efficiency, colloid stability and ease of preparation. The so-synthesized AuNPs had an average diameter of 25 ± 3 nm and an extinction maximum at 524 nm, as revealed by TEM and UV-Vis spectroscopy, respectively (Supplementary Fig. S1).

**Figure 2:**
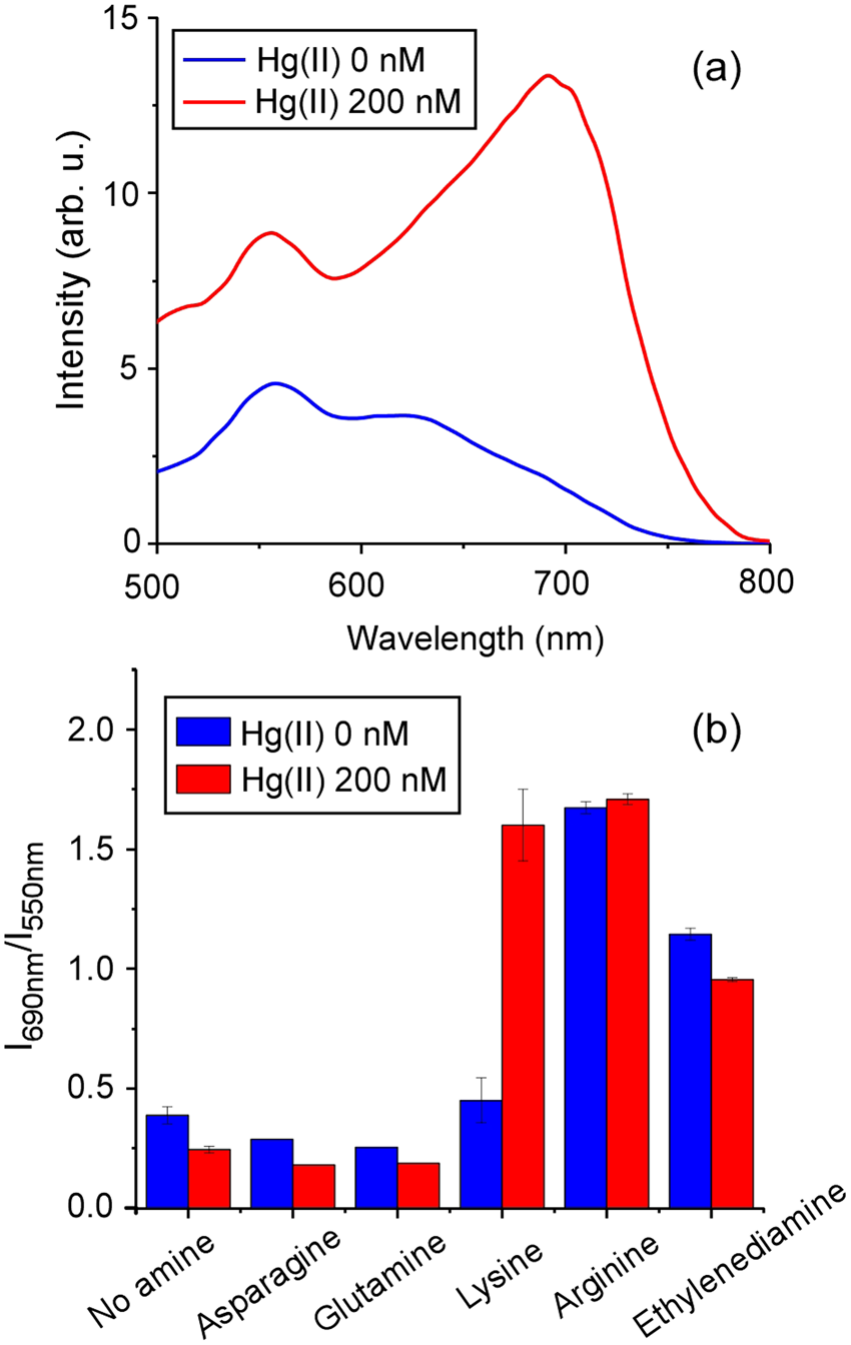
RRS response of the Hg(II) sensor. (**a**) Spectra of the sensor using 0.4 mM lysine as aggregation promoter. A clear increase of spectral intensity in the red region of the spectrum can be observed as a result of Hg(II)-induced AuNP aggregation. (**b**) Ratiometric response of the sensor using different diamines as aggregation promoters. Only lysine provided satisfactory results under the conditions evaluated. Experimental conditions: [AuNPs] = 160 pM, [phosphate buffer] = 10 mM, pH 7.0.

As a first approach, the RRS spectra of the chemical sensor (AuNPs 160 pM) in the absence and presence of Hg(II) 200 µM using lysine 0.4 mM as the aggregation promoter is shown in Fig. 2a. In the presence of Hg(II), a clear RRS increase in the red region of the spectrum can be observed as a result of AuNP aggregation (Fig. 2a and Supplementary Fig. S2). In order to select the optimum RRS ratio to monitor the sensor, different pairs of wavelengths were evaluated. The ratio I_690nm_/I_550nm_ was found to provide the best dynamic range (Supplementary Fig. S3) and thus it is used throughout the paper to quantify the Hg(II)-induced response of the sensor.

We first sought to determine whether a simple diamine such as ethylenediamine or other amino acids with an amine moiety on their side chain, could promote aggregation of the AuNPs in the presence of Hg(II), similarly or more efficiently than lysine. For this reason, experiments were carried out adding 0.4 mM of ethylenediamine, lysine, arginine, glutamine, and asparagine. As shown in Fig. 2b, the only amino acid that yielded satisfactory Hg(II) sensing results turned out to be lysine. The other tested diamines can be classified in two groups: those that induced aggregation (ethylenediamine and arginine) and those that did not (glutamine and asparagine) regardless of the presence of Hg(II). The observed behavior is consistent with a previous study of amino acid-induced AuNP aggregation^35^. Interestingly, when the diamine concentration was reduced 10-fold, down to 0.04 mM, arginine did promote aggregation in the presence of Hg(II) while lysine did not (Supplementary Fig. S4). However, it was observed that Hg(II)-induced aggregation promoted by lysine showed higher intra- and inter-day reproducibility than that promoted by arginine (Supplementary Fig. S5). Consequently, lysine 0.4 mM was used for all subsequent experiments.

**Figure 3:**
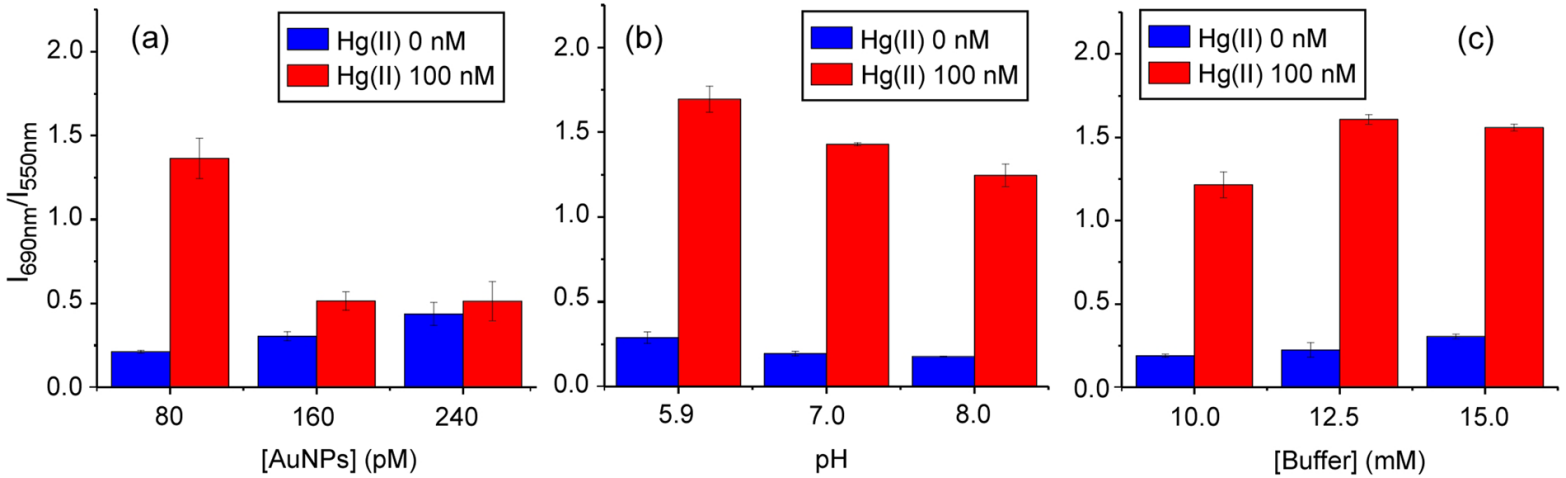
Influence of experimental conditions on the Hg(II) sensor performance. Ratiometric RRS response of the sensor for different (**a**) concentration of AuNPs, (**b**) pH’s and (**c**) phosphate buffer concentrations. Experimental conditions: (**a**) [phosphate buffer] = 10 mM, pH 7.0; (**b**) [AuNPs] = 80 pM, [phosphate buffer] = 10 mM; (**c**) [AuNPs] = 80 pM, pH 7.0.

A number of additional assay parameters were studied in order to optimize the sensor response (Fig. 3). The colloid concentration used in the assay was found to play an important role in the sensitivity of the sensor, as shown in Fig. 3a. Lowering the colloid concentration decreased aggregation of the blank and maximized the response when the analyte was present, presumably because of a more efficient coverage of the AuNPs’ surface by Hg. No further colloid concentration reduction was attempted so as not to reduce the available scattered light; thus, the concentration was kept at 80 pM for the rest of the experiments. The sensor response was also pH-dependent and a gradual increase in Hg(II)-induced aggregation was observed from pH 8.0 to pH 5.9, as shown in Fig. 3b. However, at pH 5.9, stability diminished in the absence of Hg(II) and reproducibility of different colloid preparations was compromised (Supplementary Fig. S6). These observations can be explained by the fact that lower pH’s decrease interparticle electrostatic repulsion as a result of protonation of stabilizing citrate molecules (citrate highest pK_a_ = 6.4) adsorbed on the surface of the AuNPs^36^. Because of the previous observations, all subsequent experiments were carried out at pH 7.0. Furthermore, the response of the sensor improved when the concentration of phosphate buffer was increased from 10 to 12.5 mM. Additional increases did not improve the Hg(II)-induced response, but only elevated the response of the blank (Fig. 3c). Therefore, the buffer concentration was maintained at 12.5 mM for the remaining experiments. The higher aggregation observed with increasing buffer concentrations is most likely due to the associated rise in the ionic strength of the sample, which screens more efficiently the electrostatic repulsion between particles^35,36^.

**Figure 4:**
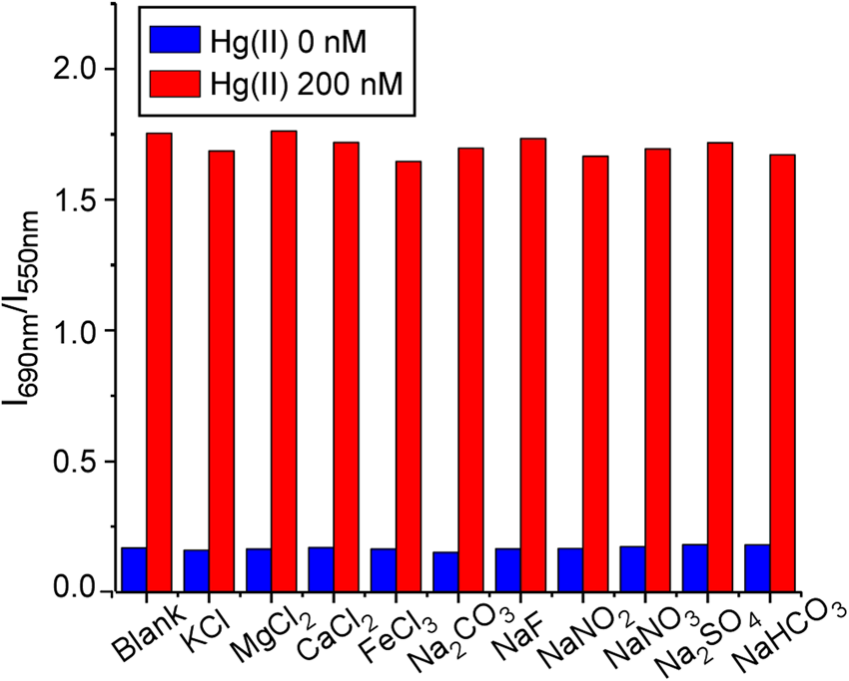
Selectivity of the sensor. The response of the sensor in the presence of some of the ions commonly present in drinking and fresh waters. The interfering salt concentration was 250 times higher than that of Hg(II).

Finally, the interference of ions commonly present in drinking and fresh waters were evaluated. In order to investigate positive and negative interferences, experiments were carried out in the absence and presence of Hg(II) with an added interferent salt at 250-fold higher concentration. The results, shown in Fig. 4, revealed good selectivity against the ions tested (K^+^, Mg^2+^, Ca^2+^, Fe^3+^, Na^+^, CO_3_^2−^, HCO_3_^−^, F^–^, Cl^−^, NO_2_^−^, NO_3_^−^ or SO_4_^2−^). Some other metallic cations that may be found as traces in drinking and fresh waters (Cd^2+^, Cu^2+^, Cr^3+^, Pb^2+^, Al^3+^, Co^2+^, Ni^2+^ and Zn^2+^) were also evaluated with good results (Supplementary Fig. S7), which strengthen the applicability of the sensor.

### LED dual-detector photometer and LED-spectral sensor for RRS measurements

**Figure 5:**
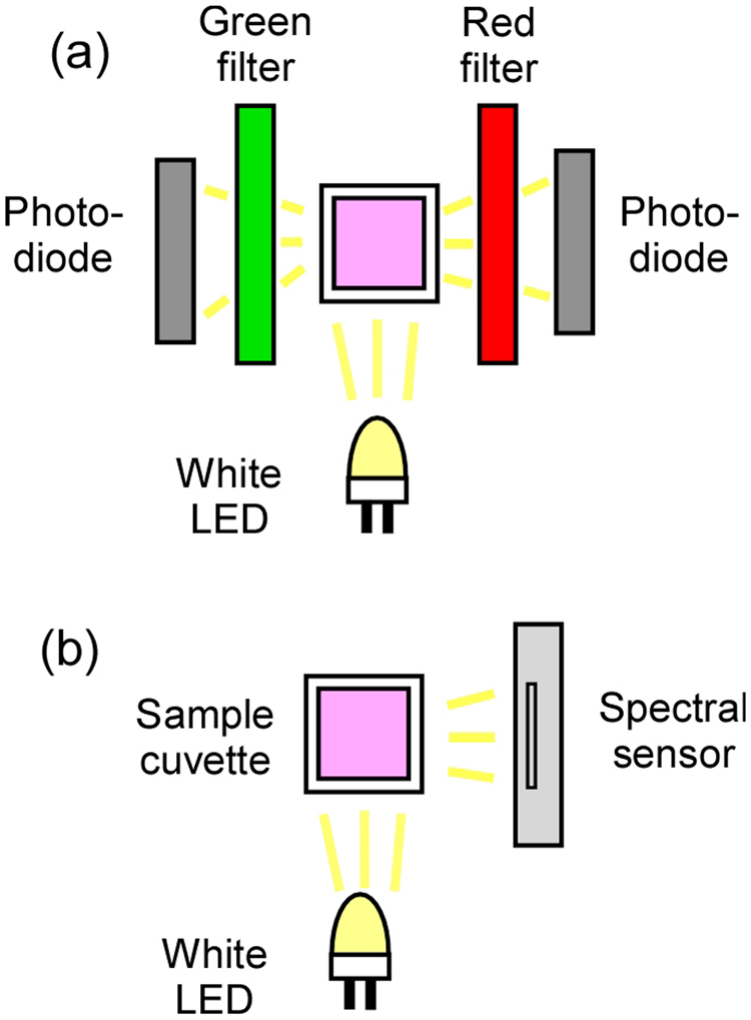
Portable devices for ratiometric RRS measurements. (**a**) LED dual-detector photometer and (**b**) LED-spectral sensor.

Once the chemical sensor was properly characterized, we sought to implement it using two simple and compact portable devices. In both of them, the sample is directly illuminated by a warm-white LED (Fig. 5 and Supplementary Fig. S8). In the first device, a LED dual-detector photometer suggested elsewhere^11^, the scattered LED light isolated by one green and one red color filters was measured by two photodiodes, as shown in Fig. 5a. The second device, shown in Fig. 5b, is based on a novel ultra-compact spectral sensor as the detector element. This sensor uses a solid-state optical component instead of the traditional diffraction grating present in spectrometers. The spectral sensor is able to acquire spectra across the entire visible region and is ideal for integration into handheld and portable devices.

**Figure 6:**
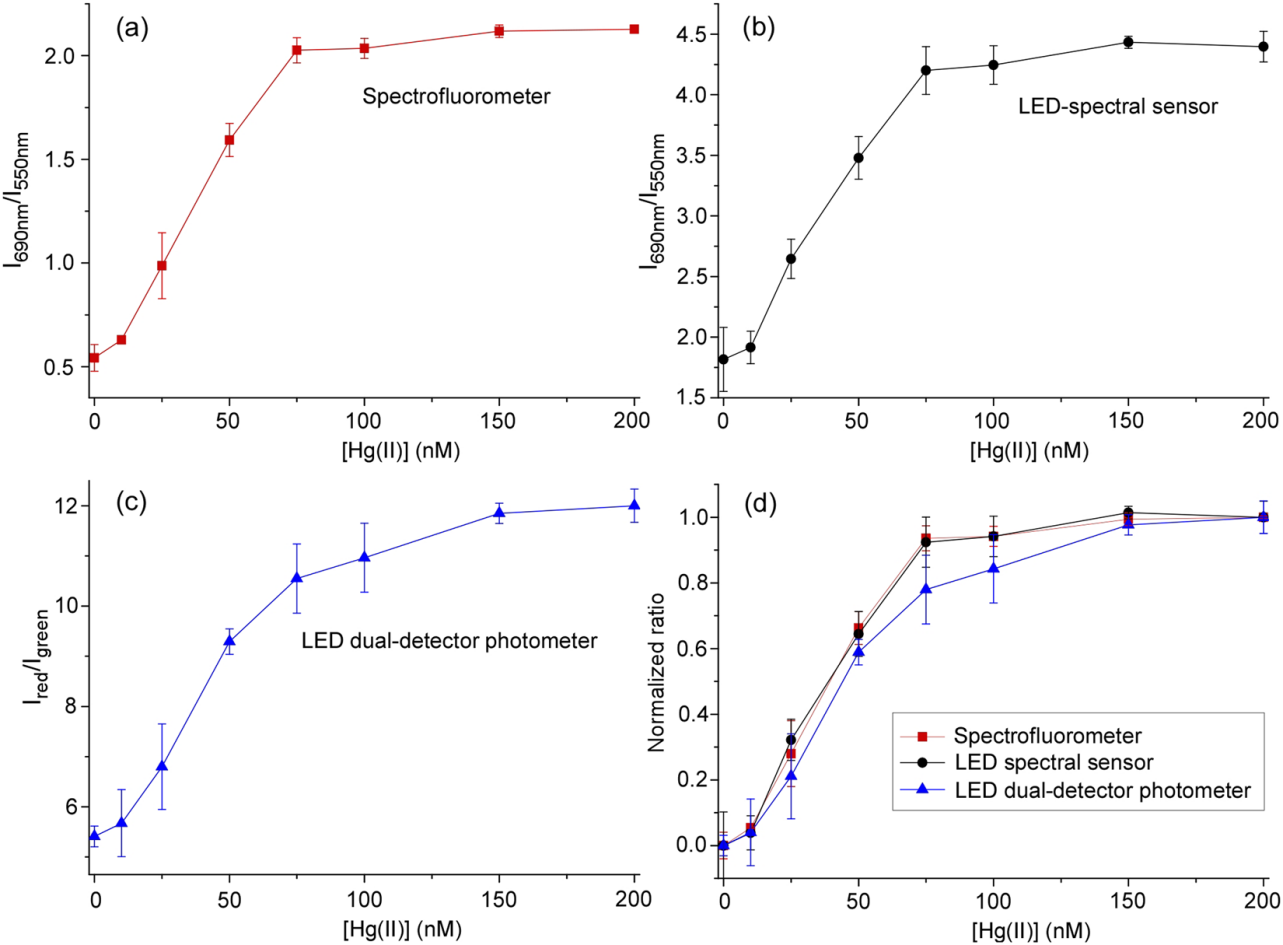
Calibration curves of the sensor measured with different instruments. Curves were acquired using the (**a**) spectrofluorometer, (**b**) LED-spectral sensor and (**c**) LED dual-detector photometer. Normalized calibration curves are shown in (**d**). The normalized ratios correspond to *(R* − *R*_0_*)/(R*_*max*_ − *R*_0_) where *R* = *I*_*690nm*_*/I*_*550nm*_.

Calibration curves of the chemical sensor in the 0–200 nM range were obtained using a high-end benchtop scanning spectrofluorometer (used for the optimization of the sensor parameters) and both portable devices, as shown in Fig. 6a–c. A linear correlation existed between the scattering ratio and the concentration of Hg(II) in the range 10–75 nM for the spectrofluorometer (R^2^ = 0.994), the LED-spectral sensor (R^2^ = 0.989), and the LED dual-detector photometer (R^2^ = 0.982). The experimental limits of detection (LODs) at a signal-to-noise ratio of 3 (3σ rule) correspond to 25 nM for the three instruments, an observation which indicates that instrumental noise from the portable devices does not limit the sensitivity of the chemical sensor. Interestingly, the attained LOD is below the maximum acceptable level of Hg(II) in drinking water (30 nM) set by the World Health Organization (WHO)^10^.

Although inconsequential for the analytical implementation of the chemical sensor, notice that the absolute ratios measured in the different instruments are not the same since these values are influenced by the wavelength-dependent excitation and detection efficiencies. In particular, the spectra and ratios measured by the LED-spectral sensor are weighed by the white LED spectrum (Supplementary Fig. S9).

To facilitate a visual comparison of calibration curves taken with all the three instruments, curves were normalized to the range [0, 1], as shown in Fig. 6d. This comparison reveals that the spectrofluorometer and LED-spectral sensor calibration curves are essentially indistinguishable, but that the LED dual-detector photometer curve exhibits a rather different saturation behavior. This is because the photometer monitors I_red_/I_green_, the ratio between the red and green region of the scattering spectrum. The specific shape of the calibration curve in this device is a function of the transmission profile of the optical filters used. Although narrow-band filters (notch type) could be used to isolate particular wavelengths of the spectrum and thus obtain specific wavelength ratios, broadband filters were selected here in order not to dramatically reduce the amount of scattered light reaching the photodiodes.

**Figure 7:**
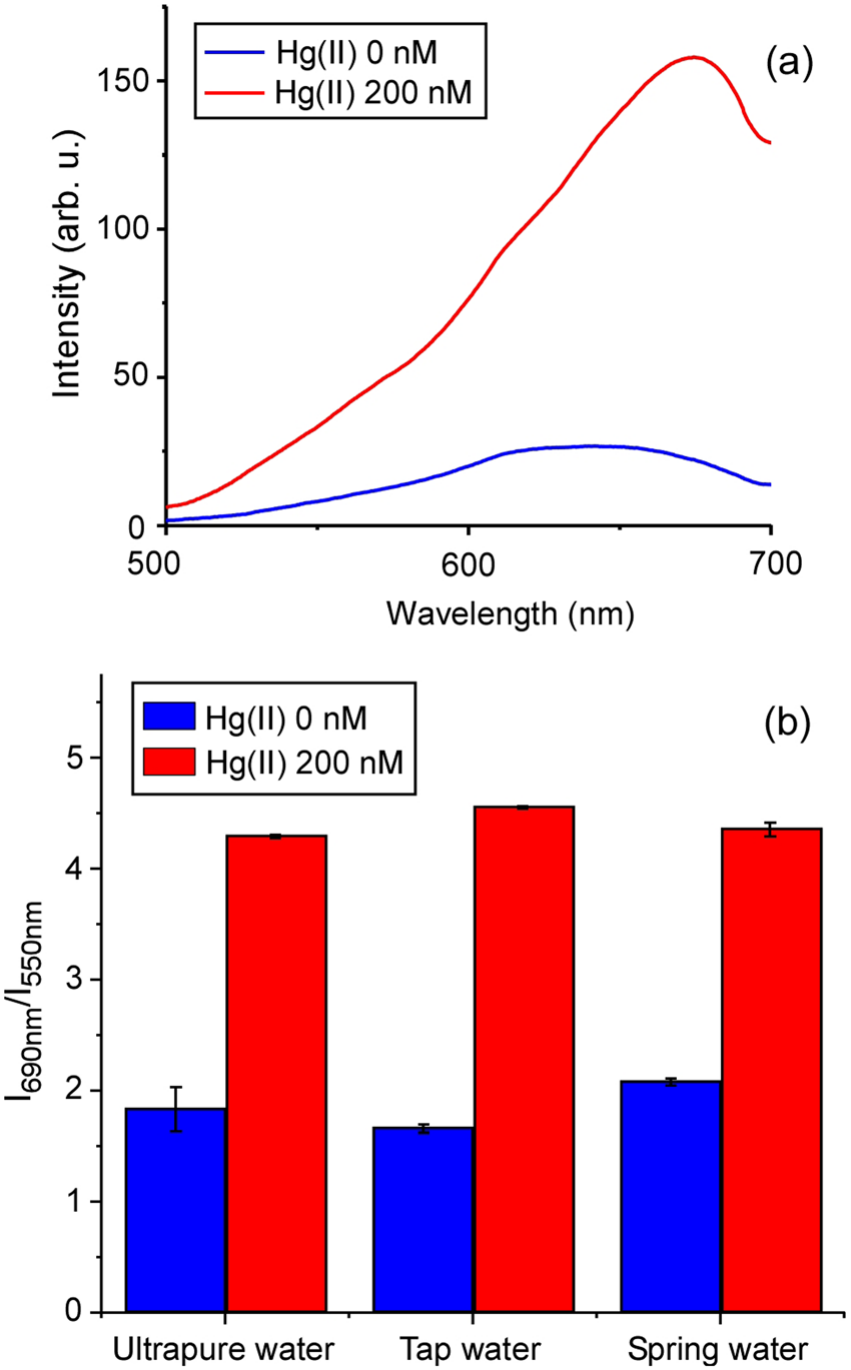
Hg(II) detection in complex matrices with the LED-spectral sensor. (**a**) Typical RRS spectra in the absence and presence of Hg(II) measured with the LED-spectral sensor. (**b**) Corresponding ratiometric RRS responses in ultrapure, tap and spring water.

As a practical application, the performance of the chemical sensor was tested in tap and spring water spiked with Hg(II) using the LED-spectral sensor. The results, shown in Fig. 7, demonstrate successful detection of the analyte despite the presence of the interfering species present in the samples (salts, organic contaminants, etc.).

## Discussion

Chemical sensors based on the aggregation of metallic nanoparticles, such as the one described here, offer a great potential in a number of practical applications^5,9,37^. In contrast to traditional analytical techniques for heavy metal quantification, aggregation-based nanosensors offer distinct advantages including rapid analysis times and the use of simple instrumentation. Most frequently, a colorimetric (extinction) strategy is adopted, in which the sensor signal is monitored with a benchtop visible spectrophotometer. A number of such plasmonic nanosensors with varied analytical capabilities has been reported in the literature for the quantification of Hg(II). The reader is referred to timely comprehensive reviews for a comparison of such approaches^9,29^. Unfortunately, robust portable implementations of aggregation-based colorimetric nanosensors for heavy metal quantification in general and for Hg(II) in particular are scarce^27^. Therefore, miniaturized portable devices able to robustly monitor the response of these plasmonic sensors are urgently needed for on-site sample analysis.

RRS is a scattering process that is highly sensitive to the aggregation state of the system. The capability to employ RRS for monitoring chemical sensors based on the aggregation of metallic colloids and the potential simplicity of the required instrumentation have been early recognized^11^. Recently, a 532-nm laser coupled to a smartphone camera was suggested as a portable RRS monitoring device for chemical sensors based on the aggregation of AuNPs^38^. However, the former device can only monitor RRS at a single wavelength and is thus unsuitable for wavelength-ratiometric sensors, such as the one described here. The response of a ratiometric sensor relies on the ratio between RRS intensities at two spectral regions and is thus intrinsically more robust (see Introduction). Consequently, we sought to investigate the performance of two low-cost portable devices able to monitor the ratiometric response of our RRS Hg(II) nanosensor and compare it with that obtained using a high-end benchtop scanning spectrofluorometer.

Regarding the analytical performance of the different instruments for monitoring the sensor response, the spectrofluorometer did not provide significant advantages in terms of LOD, dynamic range, acquisition time or selectivity. The intrinsic higher sensitivity of the benchtop instrument did not mark a difference due to the high intensity of the RRS light from the colloid. Considering that larger AuNPs and AgNPs exhibit even higher scattering efficiencies^11,17^, it is expected that most RRS sensors based on these nanoparticles could be implemented using the portable devices presented here. Acquisition times were shorter for the LED-spectral sensor (2.5 s) compared with the benchtop scanning instrument (~30 s), while readings from the LED dual-detector photometer were obtained in real time. The selectivity of the RRS sensor response is independent on the instrumentation used (spectrofluorometer or portable LED devices) as this selectivity is based on the nanosensing strategy itself, i.e. the Hg(II)-induced aggregation of AuNPs in the presence of lysine and its resulting change in RRS.

The portable devices presented here differ in versatility and ease of construction. The LED-spectral sensor allows acquiring a full scattering spectrum and thus monitoring RRS ratios at different wavelengths without any change in the optical setup. While the spectral sensor detection is currently limited to 380–700 nm, this wavelength range is sufficient for the implementation of chemical sensors based on AuNPs and AgNPs, whose LSPR bands fall in the visible spectrum^4,7^. In contrast, measuring different wavelength ratios with the LED dual-detector photometer requires changing the optical filters. It should also be noted that the spectral sensor includes all required electronics and is a plug-and-play device, while construction of the photodiode circuitry requires basic knowledge of electronics.

It is also worth highlighting the cost of the portable LED-based devices described above. Most RRS sensors have been implemented using benchtop scanning instruments^12,14–16,21–26^, which are significantly more expensive than the spectral sensor ($500). Even entry-level grating-based CCD spectrometers cost about 5 times more and are not as compact and light-weight as the spectral sensor (53 × 36 × 20 mm, 15 g). The photometer is even less expensive than the spectral sensor, but the limitations highlighted above should be taken into account.

Finally, many colorimetric plasmonic sensors reported in the literature, including those aimed at the detection of proteins, DNA, metallic ions and small molecules, could be easily adapted to an RRS scheme and implemented using low-cost portable devices such as those described above^26^. Consequently, a more widespread use of these plasmonic optical sensors integrated with portable low-cost platforms for scientific, commercial and industrial applications can be expected in the near future.

## Conclusions

A ratiometric RRS Hg(II) sensor based on AuNPs was developed and various relevant experimental parameters were studied to optimize its response. The chemical sensor was implemented in a high-end benchtop scanning spectrofluorometer and two portable low-cost devices, a LED dual-detector photometer and a LED-spectral sensor. No significant differences in the analytical performance of the chemical sensor were observed for the three instruments. The portable sensor was successfully applied for the detection of Hg(II) in complex water matrices. We believe that a number of metallic colloid RRS chemical sensors reported in the literature could take advantage of the compact devices presented here for cost-effective field-deployable applications.

## Materials and Methods

### Materials

Chloroauric acid (HAuCl_4_·3H_2_O), arginine, asparagine, glutamine and Hg(NO_3_)_2_ 0.07 M were purchased from Sigma-Aldrich, and lysine and ethylenediamine, from Merck. The rest of reagents were supplied by JT Baker. Ultrapure water was obtained with a reverse osmosis system.

### Synthesis of AuNPs

Citrate-capped AuNPs were synthesized by a nucleation and growth process reported previously^12^. Briefly, a solution of sodium citrate (50 mL; 2.2 mM) was heated to boiling under reflux and then, 333 µL of HAuCl_4_ 25 mM were added. After 30 minutes of continuous stirring, the mixture was cooled down to 90 °C and 333 µL of HAuCl_4_ 25 mM were added again and left under stirring for 30 more minutes. Then, a second identical volume of HAuCl_4_ 25 mM was added and left under stirring for 30 more minutes at 90 °C. The resulting colloid was stored at room temperature protected from light until needed.

### Characterization of AuNPs

AuNPs were characterized by transmission electron microscopy (TEM) and UV-Vis spectroscopy. For TEM measurements, an aliquot of the colloid was placed on a carbon-coated copper grid and dried at ambient temperature. TEM images were taken using a Delong America LVEM5 microscope. AuNP sizes are reported as the mean ± standard deviation of the measurements. UV-Vis spectra were acquired in a Perkin Elmer Lambda 850 spectrophotometer and used to determine the colloid concentration based on the extinction coefficient previously reported (ε_450nm_ = 1.10×10^9^ M^−1^cm^−1^)^39^.

### Hg(II) sensing assay

Hg(II) standards were prepared in 0.25% (w/w) HNO_3_ and used within a week. Unless otherwise stated, Hg(II) sensing assays were performed as follows (all concentrations refer to the final volume of the assay). Lysine or the desired diamine (0.4 mM), phosphate buffer (12.5 mM, pH 7), and Hg(II) standard (30 µL) were mixed with AuNPs (80 pM). When specified, real sample (100 µL) or an interfering salt was added (50 µM) to the mixture. In all cases, the volume was adjusted to 1.5 mL with ultrapure water. RRS measurements were carried out after 10 min of incubation.

### Benchtop spectrofluorometer RRS measurements

RRS spectra were acquired by performing a synchronous scan in a PerkinElmer LS-55 spectrofluorometer equipped with a red-sensitive photomultiplier tube (R928) using FL WinLab (PerkinElmer, Inc.). Emission correction was applied using data provided by the manufacturer. Excitation and emission slits were set to 15 and 20 nm, respectively, and the scanning speed used was 800 nm/min. RRS ratios are reported as the mean ± standard deviation of three measurements.

### LED dual-detector photometer RRS measurements

A white-LED (334-15/X1C2-1UWA, Everlight) was used as the excitation source. The LED was powered by a 5 V battery using a resistor connected in series with the LED to limit the current to 25 mA. Light scattered at 90° and 270° was measured with a pair of photodiodes. Each photodiode (PC50-7-TO8, First Sensor) was covered with a color filter to isolate the green and red regions of the RRS spectrum (FGB39 and FGL645, Thorlabs, Inc., respectively), and placed near the sample in a custom-made cuvette holder (Supplementary Fig. S8a). The signal from each photodiode was amplified using an operational amplifier (LM308N, Linear Technology) as suggested by the op-amp manufacturer (15 MΩ feedback resistors were used). The output voltages were measured with a voltmeter and the background was subtracted before analysis. RRS ratios are reported as the mean ± standard deviation of three measurements.

### LED-spectral sensor RRS measurements

A white-LED was used as the excitation source as previously described. For RRS experiments, light scattered at 90° was measured using a Spark-VIS spectral sensor (Ocean Optics, Inc.) whose entrance aperture was placed near the sample in a custom-made cuvette holder (Supplementary Fig. S8b). Spectra were averaged five times (integration time 500 ms), smoothed with a 30-point first-degree Savitzky-Golay filter and the background was substracted with OceanView (Ocean Optics, Inc.) before analysis. RRS ratios are reported as the mean ± standard deviation of three measurements.

### Real samples

The natural spring water (pH 7.8, conductivity 103 µS cm^−1^ at 25 °C) was purchased at a local store (Icelandic Glacial) and the tap water sample (pH 8.1, conductivity 593 µS cm^−1^ at 25 °C) was collected from PUCP campus.

## Electronic supplementary material

Supplementary information

## Data Availability

All data generated or analyzed during this study are included in this published article and its Supplementary Information files.
