# Supplementary material for: Portable low-cost instrumentation for monitoring Rayleigh scattering from chemical sensors based on metallic nanoparticles
Source: Sci Rep. 2018 Oct 8;8:14903. doi: 10.1038/s41598-018-33271-8 (PMC6175918; doi:10.1038/s41598-018-33271-8)
Supplement: Supplementary file 1 — Supplementary information [file 41598_2018_33271_MOESM1_ESM.pdf]

SUPPLEMENTARY INFORMATION

Portable low-cost instrumentation for monitoring Rayleigh scattering from chemical sensors based on metallic nanoparticles

Glibver Vasquez, Yulán Hernández and Yves Coello*

Departamento de Ciencias, Sección Química, Pontificia Universidad Católica del Perú PUCP, Lima, Peru.

*email: ycoello@pucp.pe Phone number: +511 626 2000 ext. 4230

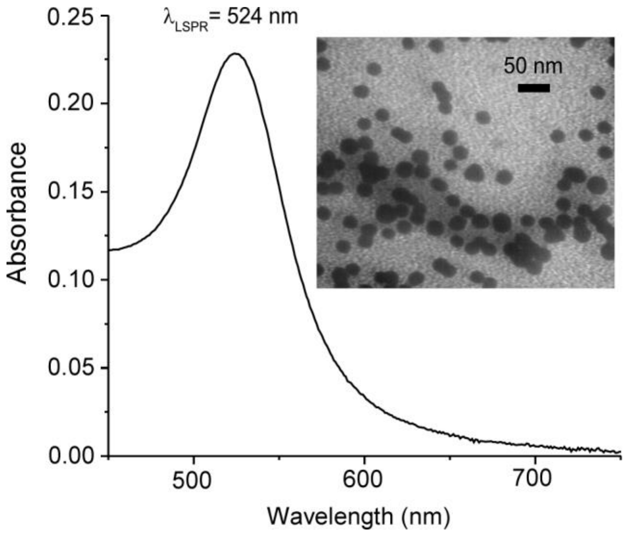

Supplementary Fig. S1. Extinction spectrum and TEM image of the synthesized AuNPs.

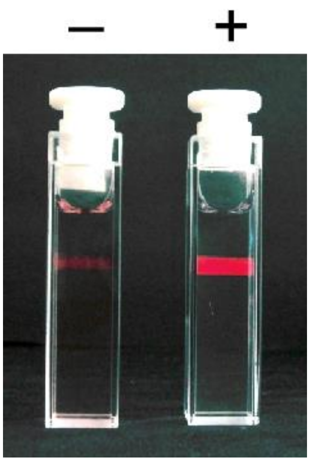

Supplementary Fig. S2. Red laser RRS from the sensor in the presence and absence of Hg(II) 200 nM.

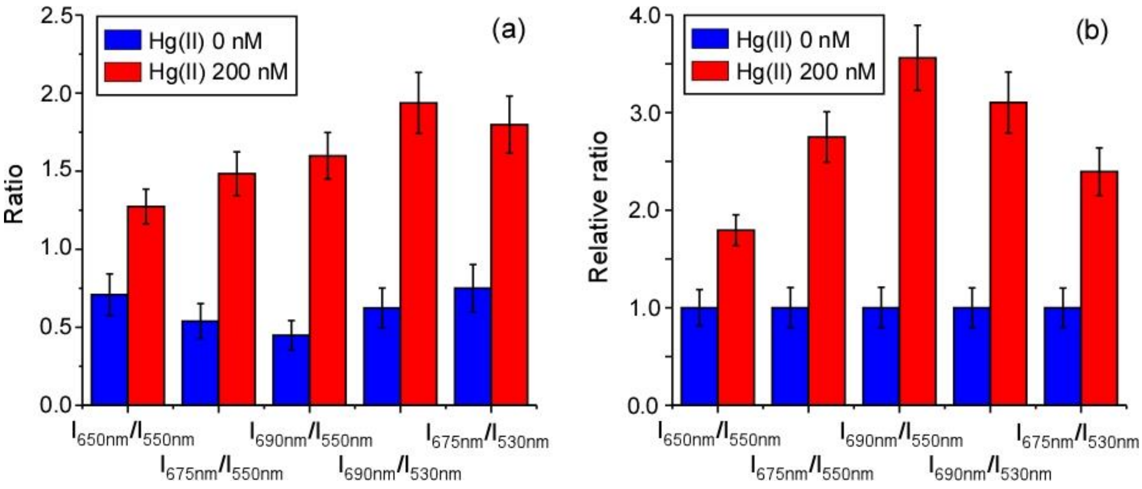

Supplementary Fig. S3. RRS response of the sensor for different wavelength ratios. Absolute and relative ratios are shown in (a) and (b), respectively. Relative ratios correspond to R/R_0 where R_0 represents the ratio for Hg(II) 0 nM.

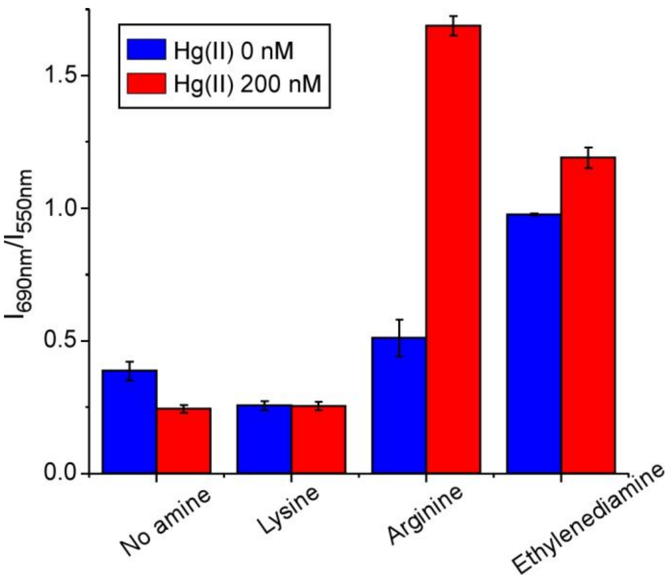

Supplementary Fig. S4. RRS response of the sensor using different diamines at low concentration (0.04 mM) as aggregation promoters.

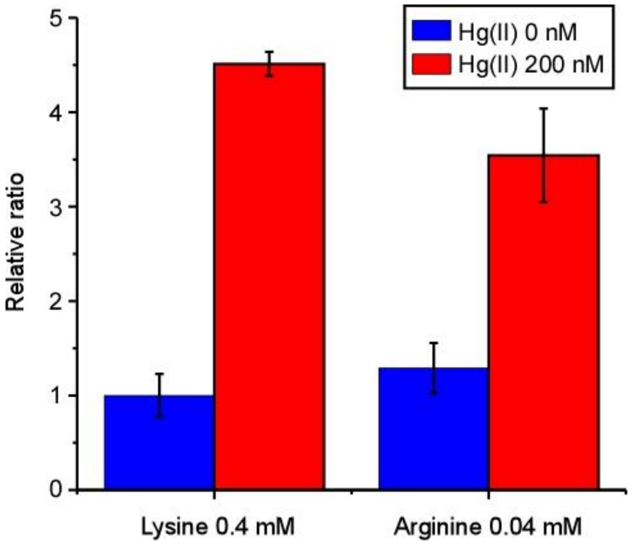

Supplementary Fig. S5. Inter-day reproducibility comparison of the RRS sensor response using lysine 0.4 mM and arginine 0.04 mM as aggregation promoters. The plot shows ratios and standard deviations of measurements taken on four different days relative to the ratio obtained for Hg(II) 0 nM using lysine 0.4 mM.

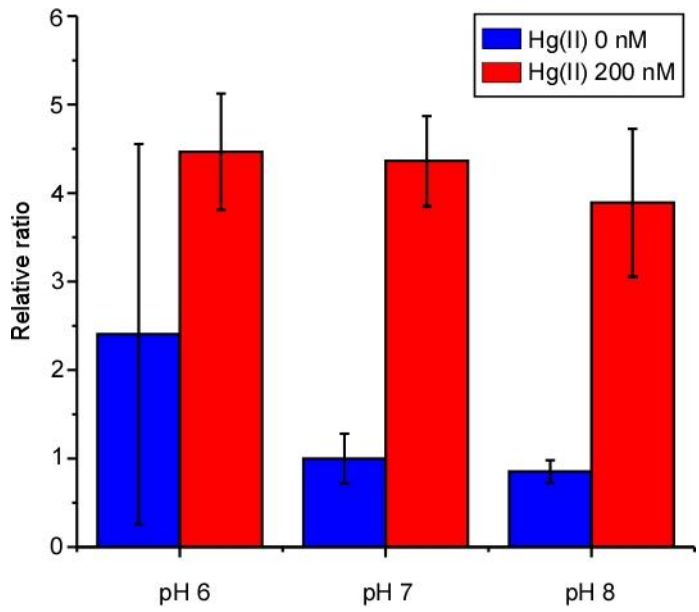

Supplementary Fig. S6. Reproducibility comparison of the RRS sensor response as a function of pH for different colloid preparations. The plot shows ratios and standard deviations of measurements taken with three different synthesized colloids relative to the ratio obtained for Hg(II) 0 nM at pH 7.

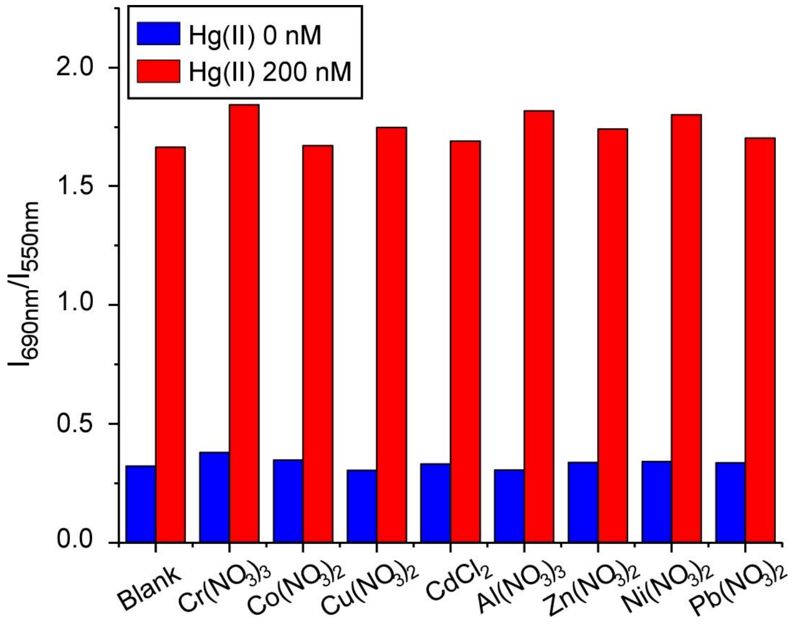

Supplementary Fig. S7. Selectivity of the sensor against metallic cations. The response of the sensor in the presence of several metallic cations that may be found as traces in drinking and fresh waters. The interfering salt concentration was 25 times higher than that of Hg(II).

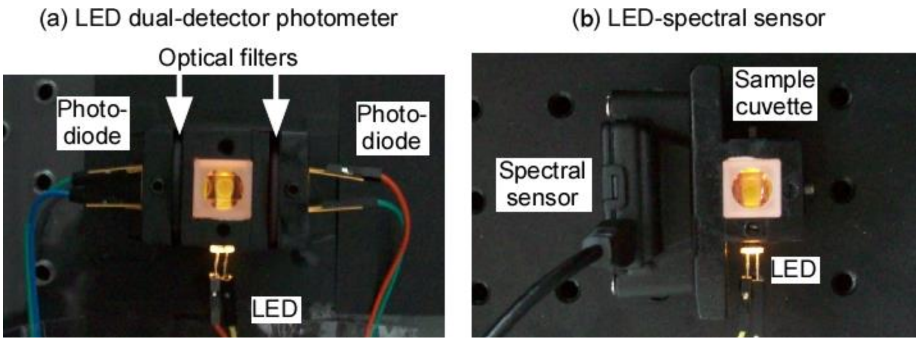

Supplementary Fig. S8. Photographs of the portable devices. (a) The sample cuvette holder used for the LED dual-detector photometer contains custom-made slots for the LED, optical filters, and photodiode detectors. Voltmeters (not shown) were used to read the signal from each photodiode. (b) In the LED-spectral sensor, the detector is directly attached to the sample cuvette holder. A laptop computer (not shown) is connected to the spectral sensor for data acquisition. Both cuvette holders were covered during measurements.

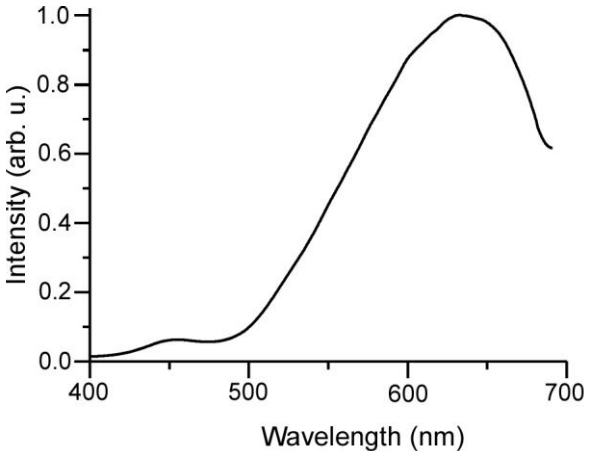

Supplementary Fig. S9. LED spectrum measured with the spectral sensor.
